# Crystal structure and Hirshfeld surface analysis of *N*-{[5-(4-methyl­phen­yl)-1,2-oxazol-3-yl]meth­yl}-1-phenyl-*N*-(prop-2-en-1-yl)methane­sulfonamide

**DOI:** 10.1107/S2056989022005035

**Published:** 2022-05-17

**Authors:** Victor N. Khrustalev, Sevim Türktekin Çelikesir, Mehmet Akkurt, Irina A. Kolesnik, Vladimir I. Potkin, Sixberth Mlowe

**Affiliations:** a Peoples’ Friendship University of Russia (RUDN University), 6 Miklukho-Maklaya St., 117198, Moscow, Russian Federation; b N. D. Zelinsky Institute of Organic Chemistry, 119991 Moscow, Leninsky prosp. 47, Russian Federation; c Department of Physics, Faculty of Sciences, Erciyes University, 38039 Kayseri, Turkey; d Laboratory of the Chemistry of Heterocyclic Compounds, Institute of Physical Organic Chemistry, National Academy of Sciences of Belarus, 13, Surganov Str., 220072, Minsk, Belarus; e University of Dar es Salaam, Dar es Salaam University College of Education, Department of Chemistry, PO Box 2329, Dar es Salaam, Tanzania; Texas A & M University, USA

**Keywords:** crystal structure, the 1,2-oxazole ring, hydrogen bonds, Hirshfeld surface

## Abstract

In the title compound, C_21_H_22_N_2_O_3_S, the 1,2-oxazole ring makes the dihedral angles of 9.16 (16) and 87.91 (17)°, respectively, with the toluene and phenyl rings, while they form a dihedral angle of 84.42 (15)° with each other. The C—S—N—C_pr_ and C—S—N—C_me_ (pr = propene, me = 3-methyl-1,2-oxazole) torsion angles are 86.8 (2) and −100.6 (3) °, respectively. In the crystal, mol­ecules are linked by C—H⋯O hydrogen bonds, generating a three-dimensional network. A Hirshfeld surface analysis was performed to investigate the contributions of the different inter­molecular contacts within the supra­molecular structure. The major inter­actions are H⋯H (53.6%), C⋯H/H⋯C (20.8%) and O⋯H/H⋯O (17.7%).

## Abstract 2

Mol­ecules in the crystal are joined together by C—H—O hydrogen bonds, forming a three-dimensional network

## 1. Chemical context

Sulfonamide anti­biotics are readily available drugs that are gradually losing their importance due to the development of bacterial resistance (Sköld, 2000 ▸). Along with the use of much less accessible anti­biotics of other classes, the design of new sulfonamides to overcome this problem seems to be reasonable (Nadirova *et al.*, 2021 ▸; Naghiyev *et al.*, 2020 ▸). One of the possible methods for structural modification is the synthesis of drug analogues containing heterocycles. From this point of view, iso­thia­zole (Kletskov *et al.*, 2020 ▸; Khalilov *et al.*, 2021 ▸) and isoxazole (Zhu *et al.*, 2018 ▸; Abdelhamid *et al.*, 2011 ▸) rings are of great inter­est. In particular, isoxazole derivatives possess a wide range of biological activity, so this heterocycle is considered to be one of the most privileged scaffolds in pharmaceutical chemistry (Altug *et al.*, 2017 ▸; Safavora *et al.*, 2019 ▸). Moreover, a lot of isoxazoles exhibit anti­bacterial properties on their own (Agrawal & Mishra, 2018 ▸; Yadigarov *et al.*, 2009 ▸), and the widely used sulfonamide anti­biotic sulfamethoxazole contains an isoxazole ring. A preliminary assessment of the biological activity of newly designed isoxazole-containing structures can be carried out *in silico* using mol­ecular docking. Data on the structural parameters of promising mol­ecules is therefore required (Gurbanov *et al.*, 2020*a* ▸,*b* ▸; Ma *et al.*, 2020 ▸,2021 ▸). All this was our motive for the synthesis and accurate structure establishment of *N*-allyl-*N*-[(5-tolyl­isoxazol-3-yl)meth­yl]benzyl­sulfonamide (**1**), which has not previously been characterized. It was obtained from isoxazolyl­allyl­amine (**2**) and benzyl sulfonyl chloride using the ‘green chemistry’ procedure developed earlier by one of us (Kolesnik *et al.*, 2022 ▸). 
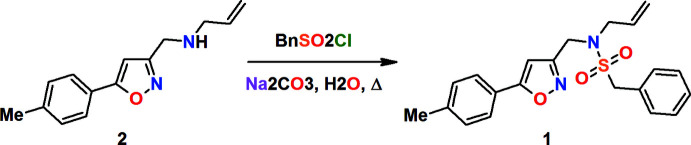


Allyl derivatives structurally similar to sulfonamide **1** are widely used as starting materials in organic synthesis for the construction of polyheterocyclic systems through intra­molecular [4 + 2] cyclo­addition reactions (Zubkov *et al.*, 2014 ▸; Krishna *et al.*, 2022 ▸).

## 2. Structural commentary

**Figure 1:**
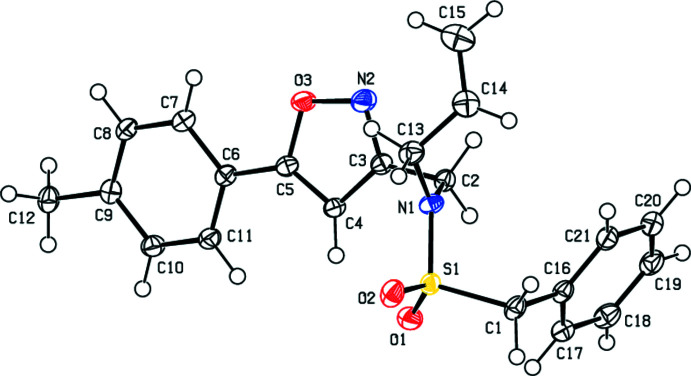
The title mol­ecule with the labelling scheme and 50% probability ellipsoids.

In the title compound (Fig. 1 ▸), the 1,2-oxazole ring (O3/N2/C3–C5) forms dihedral angles of 9.16 (16) and 87.91 (17) °, respectively, with the toluene and phenyl rings (C6–C11 and C16–C21) which subtend a dihedral angle of 84.42 (15)° with each other. The torsion angles C1—S1—N1—C2 and C1—S1—N1—C13 are 86.8 (2) and −100.6 (3) °, respectively.

## 3. Supra­molecular features and Hirshfeld surface analysis

**Figure 2:**
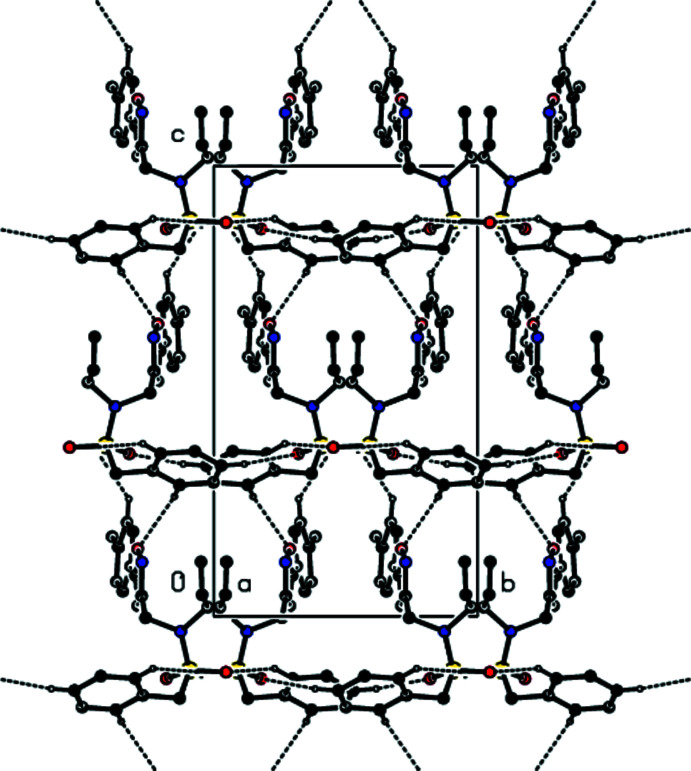
A view along the *a* axis of the C—H⋯O inter­actions in the title compound.

Mol­ecules in the crystal are joined together by C—H⋯O hydrogen bonds, forming a three-dimensional network (Table 1 ▸; Figs. 2 ▸, 3 ▸ and 4 ▸).

**Figure 5:**
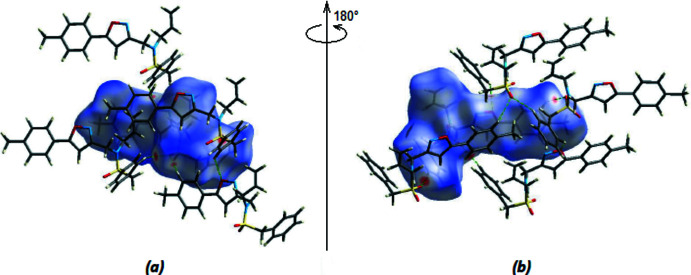
View of the three-dimensional Hirshfeld surface of the title compound plotted over *d*_norm_ in the range −0.1677 to +1.4857 a.u.

The Hirshfeld surfaces were calculated and two-dimensional fingerprint plots generated using *Crystal Explorer 17.5* (Spackman *et al.*, 2021 ▸). Fig. 5 ▸ depicts the three-dimensional Hirshfeld surface projected over *d*_norm_ in the range −0.1677 to 1.4857 a.u. The bright-red patches surrounding O1, O2, and O3 and hydrogen atoms H8, H17, H19, and H21, which highlight their activities as donors and/or acceptors, can be connected with O1, O2, and O3 inter­actions, which play a significant role in the mol­ecular packing (Tables 1 ▸ and 2 ▸).

**Figure 6:**
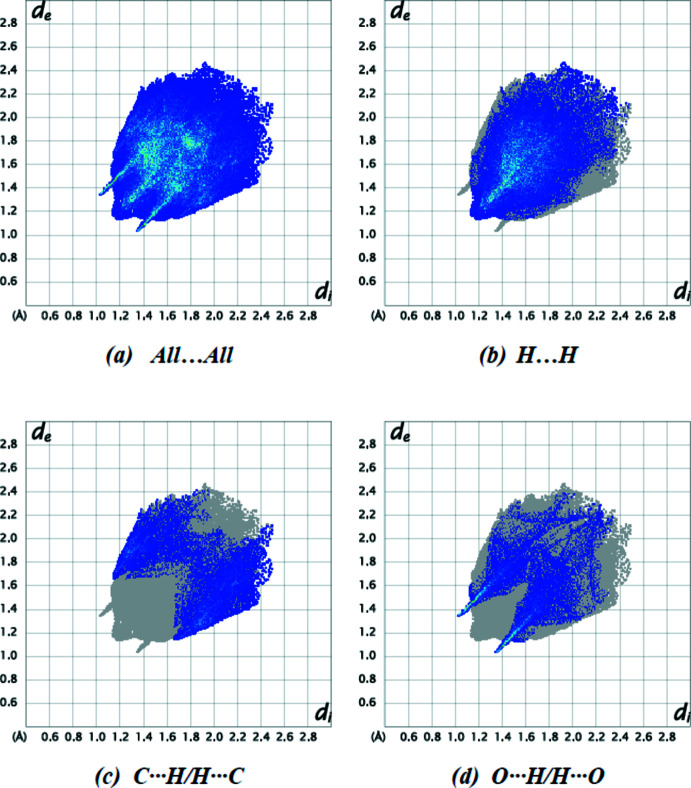
Two-dimensional fingerprint plots for the title compound, showing (*a*) all inter­actions, and delineated into (*b*) H⋯H, (*c*) C⋯H/H⋯C and (*d*) O⋯H/H⋯O inter­actions. The *d*_i_ and *d*_e_ values are the closest inter­nal and external distances (in Å) from given points on the Hirshfeld surface.

Fig. 6 ▸ *a* depicts the overall two-dimensional fingerprint plot for the title compound. The percentage contributions to the Hirshfeld surfaces from various inter­atomic inter­actions (Table 2 ▸) include H⋯H (53.6%; Fig. 6 ▸ *b*), C⋯H/H⋯C (20.8%; Fig. 6 ▸ *c*) and O⋯H/H⋯C (17.7%; Fig. 6 ▸ *d*). Other contact types, such as N⋯H/H⋯N (4.5%), C⋯C (1.7%), N⋯C/C⋯N (0.9%), and O⋯C/C⋯O (0.8%), account for less than 4.5% of the Hirshfeld surface and are likely to have little directional impact on the packing.

## 4. Database survey

Four related compounds with a methane­sulfonamide unit have been reported, *viz. N*-(4-chloro­phen­yl)-1-(5-{[(2-phen­yl­vin­yl)sulfon­yl]meth­yl}-1,3,4-oxa­diazol-2-yl)methane­sulf­on­amide (CEGKAC: Muralikrishna *et al.*, 2012 ▸), *N*-(4-flu­oro­phen­yl)methane­sulfonamide (CICPIO: Gowda *et al.*, 2007*a* ▸), *N*-(2,5-di­chloro­phen­yl)methane­sulfonamide (WIHGUQ: Gowda *et al.*, 2007*b* ▸) and *N*-(3-methyl­phen­yl)methane­sulf­on­amide (VIDKOJ: Gowda *et al.*, 2007*c* ▸).

In the crystal of CEGKAC, mol­ecules are linked by N—H⋯O hydrogen bonds, generating *C*(10) chains propagating in [001]. The packing is consolidated by C—H⋯O, C—H⋯π and very weak aromatic π–π stacking inter­actions [centroid–centroid separation = 4.085 (2) Å]. In the crystal of CICPIO, the mol­ecules are packed into a layer structure along the *a*-axis direction *via* N—H⋯O hydrogen bonds [H⋯O = 2.08 (2), N⋯O = 2.911 (6) Å and N—H⋯O = 164 (6)°]. In the crystal of WIHGUQ, the amide H atom is available to a receptor mol­ecule as it lies on one side of the plane of the benzene ring, while the methane­sulfonyl group is on the opposite side of the plane, similar to the arrangement in other methane­sulfonanilides. The mol­ecules are packed into chains through N—H⋯O and N—H⋯Cl hydrogen bonding. In the crystal of VIDKOJ, the mol­ecules are linked into chains along the *c*-axis direction through N—H⋯O hydrogen bonds.

## 5. Synthesis and crystallization

A mixture of 1,2-oxazolyl­allyl­amine **2** (1 mmol), benzyl sulfonyl chloride (1.2 mmol) and Na_2_CO_3_ (1.2 mmol) in water (15 mL) was refluxed for 4 h. After cooling, the reaction mixture was extracted with CH_2_Cl_2_ (3 × 10 mL). The combined organic fractions were washed with water (2 × 10 mL) and dried over Na_2_SO_4_. The solvent was evaporated under reduced pressure. The resulting oil was purified by flash chromatography (eluent CH_2_Cl_2_) and crystallized from MeOH as colourless crystals, yield 0.16 g (41%), m.p. 371–373 K. IR (KBr), ν (cm^−1^): 1642, 1618, 1599, 1568 (1,2-oxazole), 1343 (S=O), 1151, 1128 (SO_2_), 698 (N—SO_2_), 541 (Ar­yl). ^1^H NMR (500 MHz, CDCl_3_, 293 K): δ = 2.40 (*s*, 3H, H12*A*, H12*B*, H12*C*), 3.71–3.73 (*d*, 2H, H13*A*, H13*B*, *J* = 6.7), 4.21 (*s*, 2H, H2*A*, H2*B*), 4.33 (*s*, 2H, H1*A*, H1*B*), 5.22–5.29 (*m*, 2H, H15*A*, H15*B*), 5.63–5.71 (*m*, 1H, H14), 6.47 (*s*, 1H, H4), 7.25–7.27 (*m*, 2H, H8, H10), 7.36–7.41 (*m*, 5H, H17, H18, H19, H20, H21), 7.64–7.65 (*d*, 2H, H7, H11, *J* = 8.2). ^13^C NMR (126 MHz, CDCl_3_, 293 K): δ = 21.66, 42.55, 50.58, 59.53, 98.99, 120.50, 124.64, 125.95 (2C), 129.01 (2C), 129.06, 129.85 (2C), 130.94 (2C), 132.24, 140.86, 160.95, 170.91. MS (APCI): *m*/*z* = 383 [*M* + H]^+^.

## 6. Refinement details

Crystal data, data collection and structure refinement details are summarized in Table 3 ▸. The C-bound H atoms were positioned with idealized geometry and refined using a riding model with C—H = 0.95 Å (CH aromatic), 0.99 Å (CH_2_) and 0.98 Å (CH_3_). Isotropic displacement parameters for all H atoms were set equal to 1.2 or 1.5*U*_eq_ (parent atom). The crystal studied was refined as an inversion twin.

## Supplementary Material

Crystal structure: contains datablock(s) I. DOI: 10.1107/S2056989022005035/jy2020sup1.cif

Structure factors: contains datablock(s) I. DOI: 10.1107/S2056989022005035/jy2020Isup2.hkl

Supporting information file. DOI: 10.1107/S2056989022005035/jy2020Isup3.cml

CCDC reference: 2171930

Additional supporting information:  crystallographic information; 3D view; checkCIF report

## Figures and Tables

**Figure 3:**
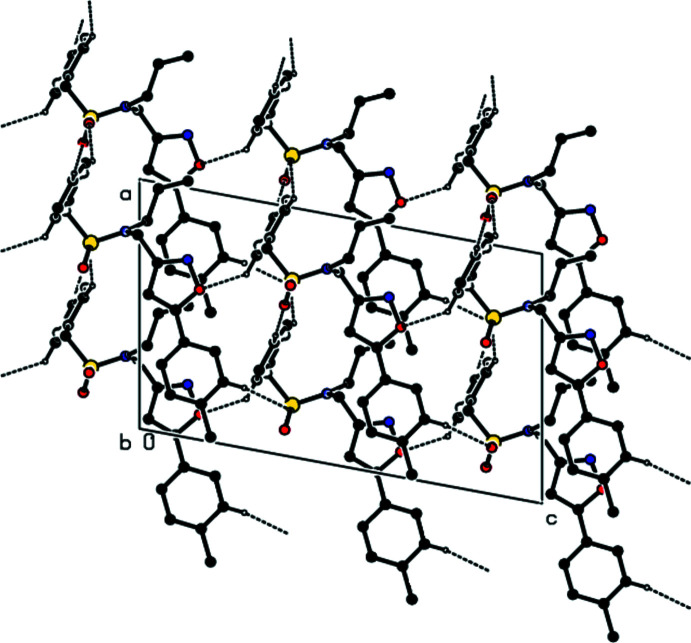
A view along the *b* axis of the C—H⋯O inter­actions in the title compound.

**Figure 4:**
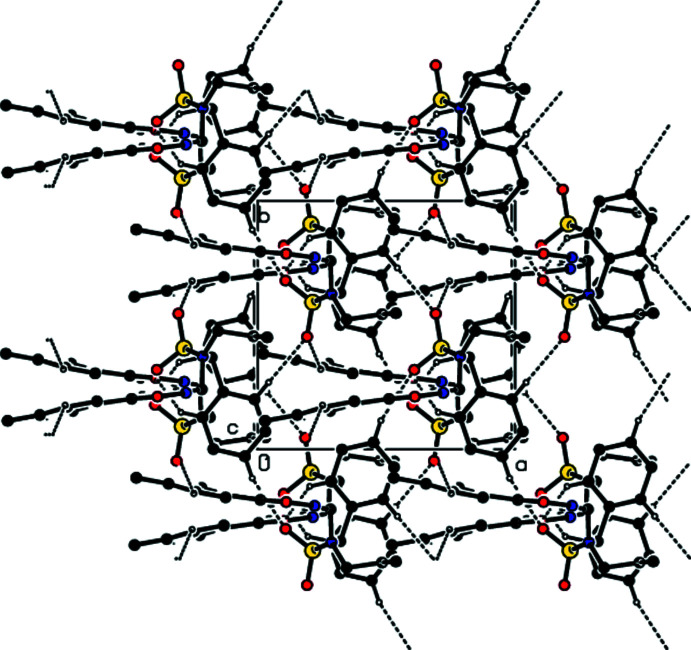
A view along the *c* axis of the C—H⋯O inter­actions in the title compound.

**Table 1:** Hydrogen-bond geometry (Å, °)

*D*—H⋯*A*	*D*—H	H⋯*A*	*D*⋯*A*	*D*—H⋯*A*
C8—H8⋯O2^i^	0.95	2.59	3.404 (4)	143
C17—H17⋯O3^ii^	0.95	2.57	3.314 (4)	135
C19—H19⋯O1^iii^	0.95	2.51	3.434 (4)	165
C21—H21⋯O2^iv^	0.95	2.50	3.369 (4)	152

Symmetry codes: (i) 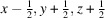 ; (ii) 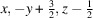 ; (iii) 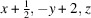 ; (iv) 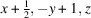 .

**Table 2:** Summary of short inter­atomic contacts (Å) in the title compound

Contact	Distance	Symmetry operation
O1⋯H19	2.51	− 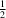 + *x*, 2 − *y*, *z*
H17⋯O3	2.57	*x*, 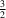 − *y*, − 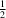 + *z*
O2⋯H21	2.50	− 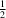 + *x*, 1 − *y*, *z*
O2⋯H8	2.59	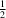 + *x*, − 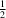 + *y*, − 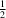 + *z*
C8⋯H18	2.92	− 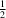 + *x*, − 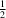 + *y*, 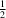 + *z*
H12*C*⋯H2*B*	2.43	−1 + *x*, *y*, *z*
C16⋯H12*B*	2.96	1 + *x*, 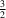 − *y*, − 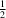 + *z*

**Table 3:** Experimental details

Crystal data	
Chemical formula	C_21_H_22_N_2_O_3_S
*M*_r_	382.46
Crystal system, space group	Monoclinic, *I* *a*
Temperature (K)	100
*a*, *b*, *c* (Å)	10.7979 (1), 10.2238 (10), 17.7316 (2)
β (°)	100.526 (1)
*V* (Å^3^)	1924.55 (19)
*Z*	4
Radiation type	Cu *K*α
μ (mm^−1^)	1.69
Crystal size (mm)	0.24 × 0.22 × 0.14

Data collection	
Diffractometer	XtaLAB Synergy, Dualflex, HyPix
Absorption correction	Multi-scan (*CrysAlis PRO*; Rigaku OD, 2021 ▸)
*T*_min_, *T*_max_	0.668, 0.779
No. of measured, independent and observed [*I* > 2σ(*I*)] reflections	21251, 3572, 3542
*R*_int_	0.051
(sin θ/λ)_max_ (Å^−1^)	0.638

Refinement	
*R*[*F* ^2^ > 2σ(*F* ^2^)], *wR*(*F* ^2^), *S*	0.045, 0.125, 1.09
No. of reflections	3572
No. of parameters	247
No. of restraints	2
H-atom treatment	H-atom parameters constrained
Δρ_max_, Δρ_min_ (e Å^−3^)	0.47, −0.58
Absolute structure	Refined as an inversion twin
Absolute structure parameter	0.00 (2)

Computer programs: *CrysAlis PRO* (Rigaku OD, 2021 ▸), *SHELXT* (Sheldrick, 2015*a* ▸), *SHELXL2018* (Sheldrick, 2015*b* ▸), *ORTEP-3 for Windows* (Farrugia, 2012 ▸) and *PLATON* (Spek, 2020 ▸).

## supplementary crystallographic information

### Crystal data

**Table:**

C_21_H_22_N_2_O_3_S	*F*(000) = 808
*M**_r_* = 382.46	*D*_x_ = 1.320 Mg m^−^^3^
Monoclinic, *I**a*	Cu *K*α radiation, λ = 1.54178 Å
*a* = 10.7979 (1) Å	Cell parameters from 18074 reflections
*b* = 10.2238 (10) Å	θ = 5.0–79.2°
*c* = 17.7316 (2) Å	µ = 1.69 mm^−^^1^
β = 100.526 (1)°	*T* = 100 K
*V* = 1924.55 (19) Å^3^	Prism, colourless
*Z* = 4	0.24 × 0.22 × 0.14 mm

### Data collection

**Table:**

XtaLAB Synergy, Dualflex, HyPix diffractometer	3542 reflections with *I* > 2σ(*I*)
Radiation source: micro-focus sealed X-ray tube	*R*_int_ = 0.051
φ and ω scans	θ_max_ = 79.6°, θ_min_ = 5.0°
Absorption correction: multi-scan (CrysAlisPro; Rigaku OD, 2021)	*h* = −13→13
*T*_min_ = 0.668, *T*_max_ = 0.779	*k* = −12→13
21251 measured reflections	*l* = −22→22
3572 independent reflections	

### Refinement

**Table:**

Refinement on *F*^2^	H-atom parameters constrained
Least-squares matrix: full	*w* = 1/[σ^2^(*F*_o_^2^) + (0.1004*P*)^2^ + 0.3109*P*] where *P* = (*F*_o_^2^ + 2*F*_c_^2^)/3
*R*[*F*^2^ > 2σ(*F*^2^)] = 0.045	(Δ/σ)_max_ < 0.001
*wR*(*F*^2^) = 0.125	Δρ_max_ = 0.47 e Å^−^^3^
*S* = 1.09	Δρ_min_ = −0.58 e Å^−^^3^
3572 reflections	Extinction correction: SHELXL-2018/3 (Sheldrick, 2015b), Fc^*^=kFc[1+0.001xFc^2^λ^3^/sin(2θ)]^-1/4^
247 parameters	Extinction coefficient: 0.0023 (4)
2 restraints	Absolute structure: Refined as an inversion twin
Primary atom site location: SHELXT	Absolute structure parameter: 0.00 (2)
Hydrogen site location: inferred from neighbouring sites	

### Special details

**Table:**

Geometry. All esds (except the esd in the dihedral angle between two l.s. planes) are estimated using the full covariance matrix. The cell esds are taken into account individually in the estimation of esds in distances, angles and torsion angles; correlations between esds in cell parameters are only used when they are defined by crystal symmetry. An approximate (isotropic) treatment of cell esds is used for estimating esds involving l.s. planes.
Refinement. Refined as a two-component inversion twin.

### Fractional atomic coordinates and isotropic or equivalent isotropic displacement parameters (Å^2^)

**Table:**

	*x*	*y*	*z*	*U*_iso_*/*U*_eq_	
S1	0.20850 (6)	0.59215 (6)	0.37939 (4)	0.0181 (2)	
O1	0.1034 (2)	0.6795 (2)	0.36066 (12)	0.0253 (5)	
O2	0.1867 (2)	0.4534 (2)	0.37509 (14)	0.0271 (5)	
O3	0.1016 (2)	0.7878 (2)	0.64904 (11)	0.0248 (5)	
N1	0.2802 (2)	0.6254 (2)	0.46571 (13)	0.0183 (5)	
N2	0.2125 (3)	0.7725 (3)	0.61968 (15)	0.0259 (6)	
C1	0.3167 (3)	0.6294 (3)	0.31677 (16)	0.0206 (6)	
H1A	0.390384	0.570343	0.328837	0.025*	
H1B	0.275212	0.612336	0.263131	0.025*	
C2	0.2760 (3)	0.7586 (3)	0.49593 (15)	0.0180 (5)	
H2A	0.258277	0.820906	0.452560	0.022*	
H2B	0.359354	0.780818	0.526667	0.022*	
C3	0.1778 (3)	0.7734 (3)	0.54474 (16)	0.0178 (5)	
C4	0.0450 (3)	0.7881 (3)	0.52263 (15)	0.0179 (5)	
H4	−0.003053	0.791270	0.472112	0.022*	
C5	0.0023 (3)	0.7966 (3)	0.58978 (15)	0.0180 (5)	
C6	−0.1207 (3)	0.8145 (3)	0.61079 (15)	0.0173 (5)	
C7	−0.1342 (3)	0.8064 (3)	0.68787 (15)	0.0199 (6)	
H7	−0.063522	0.785401	0.726289	0.024*	
C8	−0.2503 (3)	0.8290 (3)	0.70805 (15)	0.0193 (5)	
H8	−0.258482	0.822084	0.760347	0.023*	
C9	−0.3554 (3)	0.8615 (3)	0.65319 (16)	0.0189 (6)	
C10	−0.3414 (3)	0.8690 (3)	0.57646 (16)	0.0214 (6)	
H10	−0.412186	0.890631	0.538263	0.026*	
C11	−0.2261 (3)	0.8453 (3)	0.55508 (16)	0.0209 (6)	
H11	−0.218664	0.850017	0.502582	0.025*	
C12	−0.4808 (3)	0.8853 (3)	0.67664 (17)	0.0235 (6)	
H12A	−0.470660	0.949937	0.718118	0.035*	
H12B	−0.512271	0.803142	0.694488	0.035*	
H12C	−0.540911	0.918217	0.632572	0.035*	
C13	0.3384 (3)	0.5231 (3)	0.51956 (18)	0.0233 (6)	
H13A	0.325804	0.436904	0.493807	0.028*	
H13B	0.295250	0.521026	0.564246	0.028*	
C14	0.4759 (3)	0.5443 (3)	0.5472 (2)	0.0264 (6)	
H14	0.528386	0.555444	0.510125	0.032*	
C15	0.5284 (4)	0.5485 (3)	0.6206 (2)	0.0334 (8)	
H15A	0.477943	0.537711	0.658813	0.040*	
H15B	0.616492	0.562376	0.635134	0.040*	
C16	0.3618 (3)	0.7690 (3)	0.32347 (15)	0.0189 (6)	
C17	0.2818 (3)	0.8703 (3)	0.29145 (17)	0.0221 (6)	
H17	0.198709	0.850949	0.265671	0.026*	
C18	0.3237 (3)	0.9990 (3)	0.29736 (18)	0.0260 (6)	
H18	0.269020	1.067490	0.275926	0.031*	
C19	0.4454 (4)	1.0278 (3)	0.33448 (18)	0.0265 (7)	
H19	0.474088	1.115850	0.338020	0.032*	
C20	0.5246 (3)	0.9281 (3)	0.36625 (19)	0.0271 (6)	
H20	0.607762	0.947754	0.391695	0.033*	
C21	0.4827 (3)	0.7986 (3)	0.36106 (17)	0.0223 (6)	
H21	0.537283	0.730553	0.383366	0.027*	

### Atomic displacement parameters (Å^2^)

**Table:**

	*U*^11^	*U*^22^	*U*^33^	*U*^12^	*U*^13^	*U*^23^
S1	0.0203 (3)	0.0186 (3)	0.0162 (3)	−0.0028 (2)	0.0055 (2)	−0.0023 (2)
O1	0.0217 (11)	0.0310 (11)	0.0225 (10)	0.0011 (8)	0.0021 (8)	0.0003 (8)
O2	0.0370 (14)	0.0212 (10)	0.0251 (10)	−0.0095 (9)	0.0107 (10)	−0.0038 (9)
O3	0.0178 (10)	0.0414 (12)	0.0150 (10)	0.0038 (8)	0.0028 (8)	−0.0014 (8)
N1	0.0245 (12)	0.0164 (10)	0.0141 (10)	0.0019 (9)	0.0032 (9)	−0.0022 (9)
N2	0.0209 (12)	0.0395 (15)	0.0182 (12)	0.0047 (11)	0.0061 (10)	−0.0024 (10)
C1	0.0271 (15)	0.0194 (12)	0.0176 (11)	−0.0009 (11)	0.0099 (11)	−0.0021 (10)
C2	0.0204 (13)	0.0169 (11)	0.0175 (12)	−0.0005 (10)	0.0055 (10)	−0.0014 (9)
C3	0.0192 (13)	0.0180 (12)	0.0165 (12)	0.0013 (9)	0.0042 (10)	−0.0010 (9)
C4	0.0193 (13)	0.0199 (11)	0.0143 (11)	0.0007 (9)	0.0022 (10)	0.0008 (9)
C5	0.0201 (14)	0.0178 (11)	0.0160 (12)	0.0011 (10)	0.0031 (10)	−0.0007 (10)
C6	0.0216 (14)	0.0150 (11)	0.0160 (12)	0.0004 (9)	0.0049 (10)	−0.0005 (9)
C7	0.0241 (14)	0.0197 (13)	0.0162 (12)	0.0014 (10)	0.0044 (10)	0.0012 (10)
C8	0.0245 (14)	0.0188 (11)	0.0158 (12)	−0.0011 (10)	0.0065 (10)	0.0002 (10)
C9	0.0213 (13)	0.0146 (12)	0.0220 (13)	0.0002 (9)	0.0073 (11)	−0.0013 (9)
C10	0.0222 (14)	0.0235 (14)	0.0181 (13)	0.0012 (11)	0.0022 (10)	0.0015 (10)
C11	0.0206 (14)	0.0259 (13)	0.0164 (12)	0.0007 (10)	0.0042 (10)	0.0000 (11)
C12	0.0223 (15)	0.0256 (13)	0.0247 (14)	0.0003 (11)	0.0105 (12)	−0.0012 (12)
C13	0.0274 (15)	0.0190 (13)	0.0229 (14)	0.0014 (10)	0.0030 (11)	0.0044 (10)
C14	0.0257 (16)	0.0237 (14)	0.0297 (15)	0.0047 (11)	0.0046 (13)	0.0013 (12)
C15	0.0347 (18)	0.0248 (15)	0.0369 (18)	0.0049 (13)	−0.0035 (14)	−0.0022 (13)
C16	0.0235 (14)	0.0192 (13)	0.0156 (12)	0.0001 (10)	0.0079 (10)	−0.0015 (9)
C17	0.0242 (14)	0.0244 (14)	0.0175 (11)	−0.0017 (12)	0.0036 (11)	0.0006 (11)
C18	0.0360 (18)	0.0213 (13)	0.0212 (14)	0.0011 (12)	0.0061 (12)	0.0029 (11)
C19	0.0388 (19)	0.0216 (13)	0.0199 (13)	−0.0089 (12)	0.0076 (13)	0.0001 (10)
C20	0.0276 (16)	0.0323 (16)	0.0218 (14)	−0.0082 (13)	0.0055 (12)	0.0018 (12)
C21	0.0220 (14)	0.0255 (13)	0.0204 (13)	0.0013 (11)	0.0068 (11)	0.0033 (11)

### Geometric parameters (Å, º)

**Table:**

S1—O1	1.434 (2)	C9—C12	1.507 (4)
S1—O2	1.438 (2)	C10—C11	1.388 (4)
S1—N1	1.620 (2)	C10—H10	0.9500
S1—C1	1.794 (3)	C11—H11	0.9500
O3—C5	1.360 (3)	C12—H12A	0.9800
O3—N2	1.399 (3)	C12—H12B	0.9800
N1—C2	1.468 (3)	C12—H12C	0.9800
N1—C13	1.477 (4)	C13—C14	1.492 (5)
N2—C3	1.313 (4)	C13—H13A	0.9900
C1—C16	1.506 (4)	C13—H13B	0.9900
C1—H1A	0.9900	C14—C15	1.322 (5)
C1—H1B	0.9900	C14—H14	0.9500
C2—C3	1.494 (4)	C15—H15A	0.9500
C2—H2A	0.9900	C15—H15B	0.9500
C2—H2B	0.9900	C16—C21	1.387 (4)
C3—C4	1.424 (4)	C16—C17	1.401 (4)
C4—C5	1.355 (4)	C17—C18	1.389 (4)
C4—H4	0.9500	C17—H17	0.9500
C5—C6	1.455 (4)	C18—C19	1.389 (5)
C6—C11	1.400 (4)	C18—H18	0.9500
C6—C7	1.403 (3)	C19—C20	1.383 (5)
C7—C8	1.385 (4)	C19—H19	0.9500
C7—H7	0.9500	C20—C21	1.397 (4)
C8—C9	1.393 (4)	C20—H20	0.9500
C8—H8	0.9500	C21—H21	0.9500
C9—C10	1.398 (4)		
			
O1—S1—O2	119.14 (15)	C10—C9—C12	121.4 (3)
O1—S1—N1	108.03 (13)	C11—C10—C9	121.2 (3)
O2—S1—N1	107.60 (13)	C11—C10—H10	119.4
O1—S1—C1	107.57 (14)	C9—C10—H10	119.4
O2—S1—C1	107.18 (14)	C10—C11—C6	120.1 (3)
N1—S1—C1	106.70 (14)	C10—C11—H11	120.0
C5—O3—N2	109.1 (2)	C6—C11—H11	120.0
C2—N1—C13	117.3 (2)	C9—C12—H12A	109.5
C2—N1—S1	119.87 (19)	C9—C12—H12B	109.5
C13—N1—S1	122.40 (19)	H12A—C12—H12B	109.5
C3—N2—O3	105.7 (2)	C9—C12—H12C	109.5
C16—C1—S1	112.94 (19)	H12A—C12—H12C	109.5
C16—C1—H1A	109.0	H12B—C12—H12C	109.5
S1—C1—H1A	109.0	N1—C13—C14	112.9 (3)
C16—C1—H1B	109.0	N1—C13—H13A	109.0
S1—C1—H1B	109.0	C14—C13—H13A	109.0
H1A—C1—H1B	107.8	N1—C13—H13B	109.0
N1—C2—C3	112.2 (2)	C14—C13—H13B	109.0
N1—C2—H2A	109.2	H13A—C13—H13B	107.8
C3—C2—H2A	109.2	C15—C14—C13	123.4 (3)
N1—C2—H2B	109.2	C15—C14—H14	118.3
C3—C2—H2B	109.2	C13—C14—H14	118.3
H2A—C2—H2B	107.9	C14—C15—H15A	120.0
N2—C3—C4	111.5 (3)	C14—C15—H15B	120.0
N2—C3—C2	118.9 (3)	H15A—C15—H15B	120.0
C4—C3—C2	129.6 (3)	C21—C16—C17	119.3 (3)
C5—C4—C3	104.6 (2)	C21—C16—C1	120.5 (3)
C5—C4—H4	127.7	C17—C16—C1	120.2 (3)
C3—C4—H4	127.7	C18—C17—C16	120.1 (3)
C4—C5—O3	109.2 (3)	C18—C17—H17	120.0
C4—C5—C6	134.8 (3)	C16—C17—H17	120.0
O3—C5—C6	116.0 (2)	C19—C18—C17	120.3 (3)
C11—C6—C7	119.0 (3)	C19—C18—H18	119.9
C11—C6—C5	120.7 (2)	C17—C18—H18	119.9
C7—C6—C5	120.3 (3)	C20—C19—C18	119.8 (3)
C8—C7—C6	120.1 (3)	C20—C19—H19	120.1
C8—C7—H7	119.9	C18—C19—H19	120.1
C6—C7—H7	119.9	C19—C20—C21	120.2 (3)
C7—C8—C9	121.3 (2)	C19—C20—H20	119.9
C7—C8—H8	119.3	C21—C20—H20	119.9
C9—C8—H8	119.3	C16—C21—C20	120.3 (3)
C8—C9—C10	118.3 (3)	C16—C21—H21	119.9
C8—C9—C12	120.3 (3)	C20—C21—H21	119.9
			
O1—S1—N1—C2	−28.6 (3)	O3—C5—C6—C7	−7.5 (4)
O2—S1—N1—C2	−158.5 (2)	C11—C6—C7—C8	−0.2 (4)
C1—S1—N1—C2	86.8 (2)	C5—C6—C7—C8	177.3 (3)
O1—S1—N1—C13	144.0 (2)	C6—C7—C8—C9	−0.8 (4)
O2—S1—N1—C13	14.2 (3)	C7—C8—C9—C10	1.0 (4)
C1—S1—N1—C13	−100.6 (3)	C7—C8—C9—C12	179.9 (3)
C5—O3—N2—C3	0.4 (3)	C8—C9—C10—C11	−0.3 (4)
O1—S1—C1—C16	58.5 (2)	C12—C9—C10—C11	−179.1 (3)
O2—S1—C1—C16	−172.3 (2)	C9—C10—C11—C6	−0.6 (4)
N1—S1—C1—C16	−57.3 (2)	C7—C6—C11—C10	0.8 (4)
C13—N1—C2—C3	−75.0 (3)	C5—C6—C11—C10	−176.6 (3)
S1—N1—C2—C3	98.1 (3)	C2—N1—C13—C14	−65.6 (3)
O3—N2—C3—C4	−0.4 (3)	S1—N1—C13—C14	121.6 (3)
O3—N2—C3—C2	−179.9 (2)	N1—C13—C14—C15	126.2 (3)
N1—C2—C3—N2	101.3 (3)	S1—C1—C16—C21	105.6 (3)
N1—C2—C3—C4	−78.2 (4)	S1—C1—C16—C17	−74.6 (3)
N2—C3—C4—C5	0.2 (3)	C21—C16—C17—C18	0.1 (4)
C2—C3—C4—C5	179.7 (3)	C1—C16—C17—C18	−179.7 (3)
C3—C4—C5—O3	0.0 (3)	C16—C17—C18—C19	0.5 (5)
C3—C4—C5—C6	178.8 (3)	C17—C18—C19—C20	−0.6 (5)
N2—O3—C5—C4	−0.2 (3)	C18—C19—C20—C21	0.1 (5)
N2—O3—C5—C6	−179.3 (2)	C17—C16—C21—C20	−0.6 (4)
C4—C5—C6—C11	−8.8 (5)	C1—C16—C21—C20	179.2 (3)
O3—C5—C6—C11	169.9 (2)	C19—C20—C21—C16	0.5 (5)
C4—C5—C6—C7	173.8 (3)		

### Hydrogen-bond geometry (Å, º)

**Table:**

*D*—H···*A*	*D*—H	H···*A*	*D*···*A*	*D*—H···*A*
C7—H7···O3	0.95	2.44	2.763 (4)	100
C8—H8···O2^i^	0.95	2.59	3.404 (4)	143
C13—H13*A*···O2	0.99	2.36	2.867 (4)	111
C17—H17···O3^ii^	0.95	2.57	3.314 (4)	135
C19—H19···O1^iii^	0.95	2.51	3.434 (4)	165
C21—H21···O2^iv^	0.95	2.50	3.369 (4)	152

Symmetry codes: (i) *x*−1/2, *y*+1/2, *z*+1/2; (ii) *x*, −*y*+3/2, *z*−1/2; (iii) *x*+1/2, −*y*+2, *z*; (iv) *x*+1/2, −*y*+1, *z*.
